# Knowledge and practices related to stroke prevention among hypertensive and diabetic patients attending Specialist Hospital, Sokoto, Nigeria

**DOI:** 10.11604/pamj.2018.29.63.13252

**Published:** 2018-01-22

**Authors:** Sarafadeen Adeniyi Arisegi, Kehinde Joseph Awosan, Mansur Oche Oche, Anas Ahmad Sabir, Mohammed Taofeek Ibrahim

**Affiliations:** 1 Department of Family Medicine, Usmanu Danfodiyo University Teaching Hospital, Sokoto, Nigeria; 2 Department of Community Health, UsmanuDanfodiyo University, Sokoto, Nigeria; 3 Department of Internal Medicine, Usmanu Danfodiyo University, Sokoto, Nigeria

**Keywords:** Stroke prevention, knowledge, practices, hypertensive and diabetic patients

## Abstract

**Introduction:**

Stroke has become a problem of public health importance worldwide. Knowledge and practices related to stroke prevention among hypertensive and diabetic patients are important in the control of the disease. In Nigeria, recent reports indicate an emerging epidemic of stroke. This study aimed to determine the knowledge and practices related to stroke prevention among hypertensive and diabetic patients in Sokoto, Nigeria.

**Methods:**

This was a cross-sectional study among 248 patients attending hypertension and diabetes clinic of Specialist Hospital, Sokoto, Nigeria, selected by systematic sampling technique. A semi-structured questionnaire was used to collect data on the research variables. Data were analyzed using IBM SPSS version 20 statistical package.

**Results:**

The mean age of respondents was 48.21 ± 15.07 years and they were predominantly females (65.7%). The respondents had good knowledge of stroke (70.3%), organs or parts of body affected by stroke (89.1%), signs or symptoms of stroke (87.0%), stroke risk factors (86.6%) and stroke prevention (90.8%). Formal education was the sole predictor of good knowledge of signs or symptoms of stroke (aOR = 3.99, 95% CI = 1.58-10.13, p = 0.004), stroke risk factors (aOR = 4.24, 95% CI = 1.68-10.67, p = 0.002) and stroke prevention (aOR = 3.45, 95% CI = 1.09-10.93, p = 0.035). Stroke prevention practices were sub-optimal and significantly associated with formal education and being employed.

**Conclusion:**

These findings suggest the need for all stakeholders to focus on both patients' education and empowerment in halting the rising burden of stroke across the globe.

## Introduction

Stroke is a worldwide health problem and a major contributor to morbidity, mortality and disability in both developing and developed countries [1]. Stroke is the third most common cause of death in the world after heart diseases and cancers and the second leading cause of cardiovascular deaths worldwide after ischemic heart disease. The World Health Organization (WHO) estimates show that about 17.3 million people died of cardiovascular diseases (CVDs) in 2012, representing 31% of all global deaths. Of these deaths, an estimated 7.4 million were due to coronary heart diseases and 6.7 million were due to stroke. Contrary to popular belief, four out of five of these deaths occurred in the low-and middle-income countries and men and women were equally affected [2, 3]. According to the Centre for Disease Control and Prevention (CDC), stroke is the leading cause of preventable disability worldwide [4]. It is a major cause of long term disability and has potential enormous emotional and socioeconomic burden for patients, their families and health services. The often long term disabilities that accompany the disease are known to have far-reaching consequences on the well-being and quality of life of stroke survivors and their caregivers [5]. In Nigeria, stroke has been reported to account for the majority of medical admissions, with 30-day case fatality rates ranging from 28 to 37% and functional disability rates as high as 60.9% [6-8]. Although most of the stroke data in the country are hospital-based due to identified challenges in conducting community-based studies, the high burden of stroke in the Nigerian population, as with populations in other developing countries, has been widely acknowledged. The resultant permanent physical, cognitive and emotional changes from stroke affliction create pressure and life-changing demands for families and caregivers of its survivor and they are the ones who often bear the brunt of long-term care of stroke survivors, thus making them more likely to experience stress, burden and psychological morbidity [9].

Epidemiological studies have indicated that a stroke does not occur at random, there are risk factors which precede stroke by many years, therefore awareness and good knowledge of these risk factors are very crucial to its prevention. The good news is the fact that 80% of premature heart attacks and strokes are believed to be preventable when necessary precautions and actions are taken [10]. Hypertension is the most important modifiable risk factor for stroke worldwide and the risk of all stroke sub-types increases with increasing blood pressure [11, 12]. Hypertension is highly prevalent in Nigeria as in other African countries and constitutes the major risk factor for stroke in the country [13-15]. Diabetes is also a modifiable risk factor for stroke; people with diabetes are believed to have a 1.5 to 3 fold risk of stroke compared to non-diabetic subjects [16]. The prevalence of diabetes has been on the increase in many developing countries including Nigeria in recent times, owning in part to growing preference for diet comprising fatty and refined carbohydrates and obesity [15]. One of the main reasons for the rise in stroke as a cause of death is patients' lack of knowledge of the risk factors involved [17]. In addition, there is lack of patients' participation in the management of the disease. This participation demands motivation, knowledge and compliance from the patients since it is a complex lifetime regimen that needs to be followed. Patients who do not have knowledge of the risk factors of stroke are less likely to engage in stroke prevention practices like controlling their blood pressure, and behavioral pattern change such as smoking cessation and consuming a low-salt diet [18]. Considering the shortage of advanced medical technologies for the care of stroke patients in Nigeria and the economic recession that is increasingly making healthcare services inaccessible to the predominantly poor populations across the country, it is reasonable to focus our attention on stroke prevention strategies. This study was conducted to assess the knowledge and practices related to stroke prevention among hypertensive and diabetics patients attending Specialist Hospital Sokoto, Nigeria. The findings from the study would be useful to policy makers, human resource managers and other stakeholders in designing appropriate strategies and interventions for halting the rising burden of stroke across the globe.

## Methods

This was a cross-sectional study among hypertensive and diabetic patients attending the hypertension and diabetes clinic of Specialist Hospital, Sokoto, Sokoto State, Nigeria, in June and July 2016. Sokoto State has twenty-three Local Government Areas with a land mass of 25,972km^2^ (10,028m^2^) and an estimated population of 4,802,298 projected for 2015 [19]. The state has a predominance of Hausa and Fulani ethnic groups, while the non-natives belong to Igbo, Yoruba and Igala ethnic groups among others. Farmers form the greater percentage of the population, while the rest are civil servants, traders, artisans and people of other occupations like tanning and dyeing. Specialist Hospital, Sokoto, is one of the two tertiary health institutions in Sokoto metropolis. It has a bed capacity of 570 and provides medical services to the residents in the metropolis, those referred from the other Local Government Areas in the state, and patients from the neighboring states (Kebbi and Zamfara) and country (Niger republic). The sample size was estimated at 248 using Fisher's formula for estimating sample size in descriptive studies [20], an 81.1% prevalence of knowledge of risk factors of stroke from a previous study [21], a precision level of 5% and an anticipated response rate of 95%. The eligible study participants were selected by systematic sampling technique. The Internal Medicine Department of the hospital runs the hypertension and diabetes clinic twice a week (Tuesdays and Thursdays), seeing an average of 200 patients per day. One in every 7 consecutive patients presenting at the clinic was selected over a period of 8 clinic days until the required sample size was obtained. If a selected patient declined participating in the study, then the next patient was considered.

A pretested and validated semi-structured, interviewer-administered questionnaire adapted from instruments used in previous studies [5, 22] was used to obtain information on study participants' socio-demographic characteristics; knowledge of stroke, its risk factors and prevention; and participants stroke prevention practices. The questionnaire was pretested on 10 patients attending hypertension and diabetes clinic of Usmanu Danfodiyo University Teaching Hospital, Sokoto (another tertiary healthcare facility in Sokoto metropolis). Modifications were made (for clarity) based on the observations made during the pretesting. Four resident doctors assisted in questionnaire administration after being trained on the conduct of survey research, the objectives of the study, selection of study participants and questionnaire administration. Institutional ethical clearance was obtained from the Ethical committee of Specialist Hospital Sokoto, Nigeria. Permission to conduct the study was obtained from the Management of the hospital and the head of Internal Medicine Department. Informed written consent was also obtained from the participants before questionnaire administration (after explaining the objectives of the study to them and assuring them of the confidentiality of the information given by them). Data were analyzed using IBM SPSS version 20 computer statistical software package. Participants' responses to the knowledge questions were scored and graded. One mark was awarded for correct response, while wrong response or non-response received no marks. Respondents that scored 50 percent or more of expected knowledge were graded as having good knowledge, while those with scores less than 50 percent of expected knowledge were graded as having poor knowledge [23]. The chi-square test was used for bivariate analysis involving categorical variables. Logistic regression analysis was used to determine the variables that predict good knowledge of stroke, stroke risk factors and its prevention. All levels of significance were set at p < 0.05.

## Results

**Socio-demographic profile of respondents**: Out of the 248 questionnaires administered, 239 were adequately completed and found suitable for analysis, giving a response rate of 96.4 percent. The ages of the respondents ranged from 21 to 91 years (mean = 48.21±15.07). Majority of the respondents were aged below 60 years (79.1%), females (65.7%), married (74.9%), practiced Islam as religion (72.8%) and had formal education (71.5%). About a third of the respondents (33.5%) were unemployed, while those in business and civil servants constitute the majority of those that were employed (Table 1).

**Table 1:** Socio-demographic profile of respondents

Variables	Frequency (%), n = 239
**Age groups (in years)**	
20 – 39	73 (30.5)
40 – 59	116 (48.6)
60 – 79	39 (16.3)
80 and above	11 (4.6)
**Sex**	
Male	82 (34.3)
Female	157 (65.7)
**Marital status**	
Single	11 (4.6)
Married	179 (74.9)
Separated	4 (1.7)
Divorced	12 (5.0)
Widowed	33 (13.8)
**Religion**	
Islam	174 (72.8)
Christianity	65 (27.2)
**Education level**	
None and Qurranic school only	68 (28.5)
Formal (primary, secondary and tertiary)	171 (71.5)
**Occupation**	
Unemployed	80 (33.5)
Artisan	17 (7.1)
Business	66 (27.6)
Civil servant	44 (18.4)
Professional	32 (13.4)

**Figure 1:**
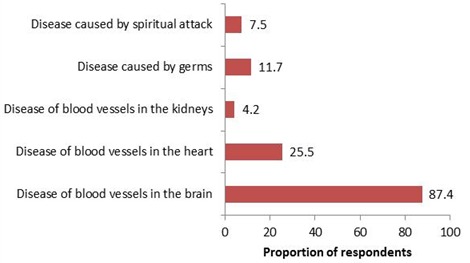
Respondents’ perception of stroke

**Respondents' perception of stroke**: Although, majority, 209 (87.4%) of the 239 respondents knew stroke as a disease of the blood vessels in the brain, about a quarter of respondents (25.5%) misperceived it as a disease of the blood vessels in the kidney, while a few also attributed it to germs (11.7%) and spiritual attack (7.5%) as shown in Figure 1.

**Figure 2:**
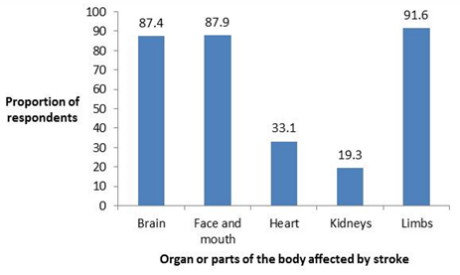
Awareness of organ or parts of the body affected by stroke among the respondents

**Awareness of organ or parts of the body affected by stroke among the respondents**: Most, 219 (91.6%) of the 239 respondents knew that stroke affects the limbs. A majority of respondents also knew that it affects the face and mouth (87.9%) and the brain (87.4%). About a third of respondents (33.1%) misconceived it to affect the heart (33.1%), while a few of them also misconceived it to affect the kidneys (19.3%) as shown in Figure 2.

**Respondents' knowledge of the signs or symptoms of stroke**: Majority, 208 (87.0%) of the 239 respondents had good knowledge of the signs or symptoms of stroke. The signs or symptoms of stroke most commonly known to the respondents include sudden and severe headache (86.2%), sudden weakness or paralysis on one side of the body (85.3%) and sudden difficulty in speaking (83.3%). Less than half of respondents (41.0%) knew sudden loss of vision as a symptom of stroke and only about a third of respondents knew that sudden loss or reduced sensation all over the body (30.5%), and sudden weakness or paralysis all over the body (38.1%) weren't symptoms or signs of stroke (Table 2).

**Table 2:** Respondents’ knowledge of signs or symptoms of stroke

Signs or symptoms of stroke	Correct response Frequency (%) n = 239
Sudden and severe headache	206 (86.2)
Sudden dizziness or loss of balance or coordination	194 (81.2)
Sudden loss of memory	143 (59.8)
Sudden loss of vision	98 (41.0)
Sudden difficulty in speaking	199 (83.3)
Sudden difficulty in swallowing	189 (79.1)
Sudden loss or reduced sensation on one side of the body	180 (75.3)
Sudden loss or reduced sensation all over the body	73 (30.5)
Sudden weakness or paralysis on one side of the body	204 (85.3)
Sudden weakness or paralysis all over the body	91 (38.1)
**Knowledge grade**	**Frequency (%)**
Good	243 (85.3)
Poor	42 (14.7)

**Respondents' knowledge of stroke risk factors**: Majority, 207 (86.6%) of the 239 respondents had good knowledge of stroke risk factors. The stroke risk factors most commonly known to the respondents include hypertension (92.0%), eating foods containing too much fat (85.7%), being overweight or obese (84.9%) and not exercising regularly (80.8%). Less than half of respondents knew aging (42.3%) and use of oral contraceptives by females (42.3%) as stroke risk factors (Table 3).

**Table 3:** Respondents’ knowledge of stroke risk factors

Stroke risk factors	Correct response Frequency (%) n = 239
Advancement in age	101 (42.3)
Not exercising regularly	193 (80.8)
Being overweight or obese	203 (84.9)
Cigarette smoking	170 (71.1)
High blood pressure (hypertension)	220 (92.0)
Diabetes mellitus	182 (76.2)
Eating foods containing too much fat	205 (85.7)
Excessive alcohol intake	174 (72.8)
Family history of stroke	131 (54.8)
Heart diseases	151 (63.2)
Use of oral contraceptives by females	101 (42.3)
Too much blood in the body (polycythemia)	66 (23.7)
**Knowledge grade**	**Frequency (%)**
Good	207 (86.6)
Poor	32 (13.4)

**Respondents' knowledge of stroke prevention**: Most, 217 (90.8%) of the 239 respondents had good knowledge of stroke prevention. The stroke prevention measures most commonly known to the respondents include ensuring appropriate treatment of hypertension (93.7%), reducing consumption of fatty foods (88.7%) and control of overweight or obesity (87.9%). Other stroke prevention measures known to the participants are as shown in Table 4.

**Table 4:** Respondents’ knowledge of stroke prevention

Stroke prevention	Correct response Frequency (%) n = 239
Engage in regular physical exercise	211 (88.3)
Lose weight (if overweight or obese)	210 (87.9)
Avoid or quit smoking	188 (78.6)
Ensure appropriate treatment of hypertension	224 (93.7)
Ensure appropriate treatment of diabetes mellitus	201 (84.1)
Reduce consumption of fatty foods	212 (88.7)
Eat fruits and vegetables regularly	208 (87.0)
Avoid excessive alcohol intake	186 (77.8)
Females should avoid use of oral contraceptives	113 (47.3)
Ensure appropriate treatment of heart diseases	171 (71.5)
Donate blood regularly	68 (28.5)
**Knowledge grade**	**Frequency (%)**
Good	217 (90.8)
Poor	22 (9.2)

**Predictors of good knowledge of signs or symptoms, risk factors and prevention of stroke among the respondents**: Although, there was significant association (p < 0.05) between good knowledge of the signs or symptoms of stroke and age below 50 years, having formal education, and being employed (Table 5), the sole predictor of good knowledge of the signs or symptoms of stroke was having formal education (Table 6). Respondents that had formal education were approximately four times likely to have good knowledge of the signs or symptoms of stroke as compared to those with none or qurranic education only (aOR = 3.99, 95% CI = 1.58-10.13, p = 0.004). Although, there was significant association (p < 0.05) between good knowledge of stroke risk factors and age below 50 years, having formal education, and being employed (Table 5), the sole predictor of good knowledge of stroke risk factors was having formal education (Table 6). Respondents with formal education were more than four times likely to have good knowledge of stroke risk factors as compared to those with none or qurranic education only (aOR = 4.24, 95% CI = 1.68-10.67, p = 0.002). Although, there was significant association (p < 0.05) between good knowledge of stroke prevention and age below 50 years, female sex, having formal education and being employed (Table 5), the predictors of good knowledge of stroke prevention were having formal education and being employed (Table 6). Respondents with formal education were approximately three times likely to have good knowledge of stroke prevention as compared to those with none or qurranic education only (aOR = 2.983, 95% CI = 1.351-6.588, p = 0.007). Similarly, respondents that were employed were more than three times likely to have good knowledge of stroke prevention as compared to those that were unemployed (aOR = 3.45, 95% CI = 1.09-10.93, p = 0.035).

**Table 5:** Distribution of knowledge of signs or symptoms, risk factors and prevention of stroke by the socio-demographic variables of respondents

Socio-demographic variables	Knowledge of signs or symptoms n = 239		Knowledge of risk factors n = 239			Knowledge of prevention n = 239		
	Good No (%)	Poor No (%)	Good No (%)	Poor No (%)		Good No (%)	Poor No (%)	
**Age (in years)**								
< 50	124 (91.2)^+^	12 (8.8)	124 (91.2)^+^	12 (8.8)		132 (97.1)^+^	4 (2.9)	
50 and above	84 (81.6)	19 (18.4)	83 (80.6)	20 (19.4)		85 (82.5)	18 (17.5)	
	c^2^= 4.808, p = 0.028		c^2^= 5.672, p = 0.017			c^2^= 14.815, p < 0.001		
**Sex**								
Male	71 (86.6)	11 (13.4)	70 (85.4)	12 (14.6)		70 (85.4)^+^	12 (14.6)	
Female	137 (87.7)	20 (12.7)	137 (87.3)	20 (12.7)		147 (93.6)	10 (6.4)	
	c^2^= 0.022, p = 0.883		c^2^= 0.167, p = 0.682			c^2^= 4.402, p = 0.036		
**Education**								
Formal	160 (93.6)^+^	11 (6.4)	160 (93.6)^+^	11 (6.4)		163 (95.3)^+^	8 (4.7)	
Non-formal	48 (70.6)	20 (29.4)	47 (69.1)	21 (30.9)		54 (79.4)	14 (20.6)	
	c^2^= 22.758, p < 0.001		c^2^= 25.080, p < 0.001			c^2^= 14.735, p < 0.001		
**Occupation**								
Employed	157 (96.1)^+^	6 (3.9)	157 (96.1)^+^	6 (3.9)		157 (96.1)^+^	6 (3.9)	
Unemployed	63 (82.8)	13 (17.2)	62 (82.2)	14 (17.8)	65 (86.8)		11 (13.2)	
	c^2^= 8.038, p = 0.005		c^2^= 8.567, p = 0.003		c^2^= 5.672, p = 0.017			

+ Statistically significant (p < 0.05)

**Table 6:** Predictors of good knowledge of signs or symptoms, risk factors and prevention of stroke among the respondents

Variables	Odds Ratio (OR)	95% CI		p value
		Lower	Upper
**Knowledge of signs or symptoms**				
Age (< 50 years versus 50 years and above)	0.659	0.271	1.602	0.358
Sex (females versus males)	1.108	0.460	2.669	0.819
Education (formal versus none/qurranic only)	3.994^+^	1.575	10.129	0.004
Occupation (employed versus unemployed)	2.249	0.574	8.818	0.245
**Knowledge of risk factors**				
Age (< 50 years versus 50 years and above)	0.645	0.268	1.556	0.329
Sex (females versus males)	0.997	0.420	2.367	0.995
Education (formal versus none/qurranic only)	4.235^+^	1.681	10.671	0.002
Occupation (employed versus unemployed)	2.273	0.579	8.916	0.239
**Knowledge of prevention**				
Age (< 50 years versus 50 years and above)	1.394	0.631	3.080	0.411
Sex (females versus males)	0.717	0.330	1.555	0.399
Education (formal versus none/qurranic only)	2.983^+^	1.351	6.588	0.007
Occupation (employed versus unemployed)	3.454^+^	1.092	10.926	0.035

+ Statistically significant (p < 0.05)

**Respondents' stroke prevention practices**: Most of the respondents observed some of the stroke prevention practices such as smoking cessation (85.4%) and reduction in alcohol intake (75.7%), while about two-thirds (66.5%) take prescribed medication for hypertension or diabetes regularly. Less than two-thirds consistently reduce their alcohol intake (58.6%), only about half of respondents (50.2%) attend follow up visits at the clinic regularly and just a few (16.7%) take prescribed drugs for heart disease regularly. Majority of respondents do home monitoring of hypertension or diabetes either rarely or not at all. Other stroke prevention practices reported by the respondents are as shown in Table 7. Compliance with stroke prevention practices was found to be associated with having formal education (X^2^ = 17.327, p < 0.001) and being employed (X^2^ = 11.658, p = 0.001).

**Table 7:** Respondents’ stroke prevention practices

Stroke prevention practices	How often (n = 239)				
	Not at all No (%)	Rarely No (%)	Sometimes No (%)	Often No (%)	Very often No (%)
Attend follow up visit at clinic	4 (1.7)	7 (2.9)	28 (11.7)	80 (33.5)	120 (50.2)
Take prescribed medications for hypertension and diabetes	1 (0.4)	4 (1.7)	16 (6.7)	59 (24.7)	159 (66.5)
Do home monitoring of blood pressure	121 (50.6)	45 (18.8)	39 (16.3)	12 (5.0)	22 (9.2)
Do home monitoring of blood sugar	171 (71.5)	17 (7.1)	30 (12.6)	11 (4.6)	10 (4.2)
Take cholesterol lowering drugs	92 (38.5)	16 (6.7)	56 (23.4)	61 (25.5)	14 (5.9)
Take low dose aspirin	44 (18.4)	16 (6.7)	33 (13.8)	70 (29.3)	76 (31.8)
Eat fruits and vegetables	13 (5.4)	4 (1.7)	24 (10.0)	98 (41.0)	100 (41.8)
Eat foods low in saturated fat	9 (3.8)	5 (2.1)	35 (14.6)	95 (39.7)	95 (39.7)
Reduce salt intake	8 (3.3)	8 (3.3)	14 (5.9)	69 (28.9)	140 (58.6)
Engage in weight control measures	11 (4.6)	10 (4.2)	39 (16.3)	91 (38.1)	88 (36.8)
Reduce alcohol intake	13 (5.4)	4 (1.7)	9 (3.8)	32 (13.4)	181 (75.7)
Avoid use of oral contraceptives (by women)	73 (30.5)	19 (7.9)	27 (11.3)	22 (9.2)	98 (41.0)
Avoid or quit smoking	14 (5.8)	2 (0.8)	2 (0.8)	17 (7.1)	204 (85.4)
Take prescribed medication for heart disease	118 (49.4)	45 (18.8)	19 (7.9)	17 (7.1)	40 (16.7)
Donate blood regularly	128 (53.6)	68 (28.5)	31 (13.0)	6 (2.5)	6 (2.5)

## Discussion

The ages of the respondents in this study ranged from 20 to 91 years with a mean age of 48.21 ± 15.07 years, this compares well with the findings in a study conducted in south-western Nigeria by Komolafe et al [24], in which the ages ranged from 16 to 95 years with a mean age of 53 ± 16 years. Another study by Wahab et al [25], also reported a mean age of 56.4 ± 12.6 years. On the contrary, a younger population with a mean age of 36.8 ± 14 years was observed in a study conducted in Ghana [26]. The preponderance of females in this study (65.7%) could be related to the fact that females have been identified to have good health-seeking behavior compared with males [27]. This is similar to the finding in a study in Uganda where 68% of the study participants were women [28] and another study by Nakibuuka et al [29], where 71.9% of the respondents from urban area and 59.6% from rural area were females. In contrast to the finding in this study, in a study done in Ghana, most of the respondents were males [26]. Unlike the poor perception of stroke reported in previous studies among patients in Nigeria [30, 31] and even in community based studies in the developed countries like United States [16] and Australia [32], majority of the respondents in this study (87.4%) knew stroke as a disease of the blood vessels in the brain. Majority of the respondents in this study were aware of the organ or parts of body affected by stroke (89.1%). Majority of them also had good knowledge of the signs or symptoms of stroke (87.0%), with sudden and severe headache and sudden weakness or paralysis on one side of the body being the most commonly known signs or symptoms of stroke. This is in agreement with the finding in a study in Ghana that reported numbness or paralysis as the commonest stroke warning sign known to respondents [26]. While it also concurs with the findings in studies done in Osogbo, Nigeria [25] and Benin, Nigeria [33], it differs from the findings in studies conducted in Australia [32] and Ireland [34], that reported visual problems and slurred speech respectively as the commonest stroke signs identified. The gaps identified in the knowledge of the signs and symptoms of stroke among the respondents in this study underscore the need for healthcare providers to give sufficient attention to educating their patients on the signs and symptoms of the disease at every clinic visit.

This would enable them seek care early enough, prevent disease progression, and avert fatal complications. Knowledge of stroke risk factors, especially identification of one's personal risk, is believed to play an important role in stoke prevention [35, 36]. This study showed good knowledge of stroke risk factors among the respondents (86.6%), with hypertension being the most commonly reported risk factor (92.0%). This finding corroborates the findings in several studies that had consistently identified hypertension as the most important modifiable stroke risk factor or cause of stroke [37-40]. In contrast to the findings in this study, poor community awareness of stroke causes or risk factors has been reported in studies conducted both in Nigeria [31, 34] and other countries across the globe including Brazil [41], Ireland [34], Pakistan [42] and the United States [43]. Although, most of the respondents in this study had good knowledge of stroke risk factors (86.6%) and stroke prevention (90.8%), the fact that only about half of them (54.8%) knew family history as a risk factor for stroke is of serious concern, as it shows that those whose first degree relatives have had stroke are unlikely to perceive themselves to be at an increased risk of the disease, or comply with stroke prevention practices. The importance of first-degree relatives of stroke survivors being aware of being at an increased risk of stroke is supported by the finding of the Lund Stroke Register study that reported higher prevalence of stroke or TIA (12.3%) among first-degree relatives of stroke patients as compared with first-degree relatives of control subjects (7.5%); Odds Ratio (OR) = 1.74, 95% Confidence Interval (CI): 1.36-2.22 [44]. In another large cohort study in China, in addition to family history of stroke being an independent risk factor for stroke, the more first-degree relatives are affected by stroke, the higher the individuals' risk of suffering from stroke [45]. Data from the Nigeria Demographic and Health Survey 2013 showed that the modern contraceptives' prevalence rate in Nigeria increased progressive from 4% in 1990 to 10% in 2013 and oral contraceptives were the second most commonly used modern contraceptive method after injectables [46]. A meta-analysis by Roach et al [47] found 1.6 fold increased risk of myocardial infarction or ischemic stroke among women on combined oral contraceptives (COCs). T

he relatively low proportion of respondents (42.3%) that knew use of oral contraceptives as a risk factor for stroke in this study is disturbing as it suggests that the increasing population of women of reproductive age on COCs in Nigeria are probably unaware of the adverse effects. The significant association between good knowledge of stroke risk factors and both having formal education and being employed (with having formal education being the sole predictor of good knowledge of stroke risk factors) among the respondents in this study is in agreement with the finding in a study by Samal et al [48], that reported that knowledge of stroke risk factors was influenced by educational level. Similar to the findings in this study, income and education were also found to be the determinants of knowledge of stroke risk factors in studies conducted in Australia [32], Brazil [41] and Ireland [34]; but this was not the case in a study conducted in Ghana [26]. The good knowledge of stroke prevention demonstrated by most of the respondents in this study (90.8%) with most of them identifying appropriate treatment of hypertensive and diabetes as preventive measures for stroke is reassuring and it is expected to facilitate adherence to treatment among those with these disease conditions. The significance of adherence to treatment was shown in the findings in studies by Neal et al [49], that reported 35-44% reduction in the incidence of stroke with appropriate treatment of hypertension and Colhoun et al [50], that reported reduction in risk of stroke with appropriate treatment of diabetes. Also, behavioral/lifestyle modifications have been shown to contribute up to 80% reduction in the risk of stroke [51]. Despite the good knowledge of stroke prevention among the respondents in this study, the practice of stroke prevention was sub-optimal among them. Only about half of respondents (50.2%) attend follow up visits at the clinic regularly, approximately two-thirds (66.5%) take prescribed medication for hypertension or diabetes regularly and just a few (16.7%) take prescribed drugs for heart disease regularly.

The poor adherence to treatment of hypertension and diabetes among the respondents in this study despite knowing the diseases to be risk factors for stroke is of serious concern as it suggests a wide gap between knowledge and practice of stroke prevention among them. Contrary to the finding in this study, another study conducted to evaluate the adherence to anti hypertensive therapy (AHT) and its implications on mortality rate and cardiovascular morbidity in a large cohort of patients in a clinical practice reported significantly lowered risk to death, stroke disabilities or acute myocardial infarction in patients with good and excellent adherence to AHT. Thus, the researchers concluded that the preliminary evidence underline the need to monitor and improve medication adherence in clinical practice [52]. The compliance by majority of the respondents in this study with behavioral/lifestyle measures for stroke prevention such as smoking cessation (85.4%), and reduction in alcohol intake (75.7%) is noteworthy in view of its benefits as reported in a study by Zhang et al [53], that found that adherence to more of elements of healthy lifestyles reduced the incidence of total, ischemic, and hemorrhagic stroke. The significant association between having formal education and compliance with stroke prevention practices in this study differs from the finding in a study in Iran [54], which reported that illiterate people surprisingly had a higher awareness of stroke and were engaged in stroke prevention practices, thus lowering stroke incidence. This finding underscores the need for policymakers, human resource managers and other stakeholders to make education of patients (particularly those with chronic diseases) an essential component of the management protocols at all levels of healthcare services provision; and it should be complemented by public health education through the mass media. Likewise, the significant association between being employed and compliance with stroke prevention practices, particularly adherence to medication, brings to the fore the need for government to alleviate poverty through job creation and provision of credit facilities for small scale enterprises; this would empower the patients to access healthcare services, facilitate compliance with stroke prevention practices, and invariably halt the rising burden of stroke across the globe.

**Limitations of the study**: Generalization of the findings of this study to the general populace is limited, being a hospital based study among patients accessing care for diseases that are closely related to stroke. The use of questionnaire to obtain information on the participants' self-reported stroke prevention practices seems not to provide enough evidence of their actual practices.

## Conclusion

Stroke prevention practices were sub-optimal despite good knowledge of stroke risk factors and prevention among the study participants. Being employed and having a formal education were the main predictors of having good knowledge of the risk factors and being compliant with the prevention strategies. These findings suggest the need for all stakeholders to focus on both patients' education and empowerment in halting the rising burden of stroke across the globe.

### What is known about this topic

- Stroke is among the leading cause of cardiovascular deaths worldwide, with approximately four-fifths of the deaths occurring in low-and medium-income countries;
- The high burden of stroke in low-and middle-income countries has been linked to the poor knowledge and rising prevalence of its risk factors (including hypertension and diabetes);
- Behavioral/lifestyle modification has been shown to contribute up to 80% reduction in the risk of stroke.

### What this study adds

- Even though the knowledge of stroke risk factors and prevention was high among hypertensive and diabetic patients in Sokoto, Nigeria, compliance with stroke prevention practices was sub-optimal;
- Knowledge and practice of stroke prevention were associated with both having formal education and being employed;
- This study highlights the importance of patients' education and empowerment in promoting good knowledge of stroke prevention and facilitating compliance with stroke prevention practices.

## Competing interests

The authors declare no competing interests.
